# Dietary intake of tocopherols and risk of incident disabling dementia

**DOI:** 10.1038/s41598-021-95671-7

**Published:** 2021-08-12

**Authors:** Shoko Aoki, Kazumasa Yamagishi, Koutatsu Maruyama, Rie Kishida, Ai Ikeda, Mitsumasa Umesawa, Cui Renzhe, Yasuhiko Kubota, Mina Hayama-Terada, Yuji Shimizu, Isao Muraki, Hironori Imano, Tomoko Sankai, Takeo Okada, Akihiko Kitamura, Masahiko Kiyama, Hiroyasu Iso

**Affiliations:** 1 Department of Public Health Medicine, Faculty of Medicine, and Health Services Research and Development Center, University of Tsukuba, Tennodai 1-1-1, Tsukuba, 305-8575 Japan; 2 Osaka Center for Cancer and Cardiovascular Disease Prevention, Osaka, Japan; 3 Ibaraki Western Medical Center, Chikusei, Japan; 4 Department of Bioscience, Graduate School of Agriculture, Ehime University, Matsuyama, Japan; 5 Department of Public Health, Juntendo University, Tokyo, Japan; 6 Department of Public Health, Dokkyo Medical University, Mibu, Japan; 7 Public Health, Department of Social Medicine, Osaka University Graduate School of Medicine, Suita, Japan; 8 Yao City Public Health Center, Yao, Japan; 9 Department of Public Health and Nursing, Faculty of Medicine, University of Tsukuba, Tsukuba, Japan; 10 Research Team for Social Participation and Community Health, Tokyo Metropolitan Institute of Gerontology, Tokyo, Japan

**Keywords:** Neurology, Risk factors

## Abstract

Tocopherols, strong antioxidants, may be useful in preventing dementia, but the epidemiological evidence is insufficient. We performed a community-based follow-up study of Japanese, the Circulatory Risk in Community Study, involving 3739 people aged 40–64 years at baseline (1985–1999). Incident disabling dementia was followed up from 1999 through 2020. For subtype analysis, we classified disabling dementia into that with and that without a history of stroke. Dietary intake of tocopherols (total, α, β, γ, and δ) were estimated using 24-h recall surveys. During a median follow-up of 19.7 years, 670 cases of disabling dementia developed. Total tocopherol intake was inversely associated with risk of disabling dementia with multivariable hazard ratios (95% confidence intervals) of 0.79 (0.63–1.00) for the highest versus lowest quartiles of total tocopherol intake (P for trend = 0.05). However, the association was strengthened when further adjusted for α-linolenic acid intake (Spearman correlation with total tocopherol intake = 0.93), with multivariable hazard ratios of 0.50 (0.34–0.74) (P for trend = 0.001) but was weakened and nonsignificant when further adjusted for linoleic acid intake (Spearman correlation with total tocopherol intake = 0.92), with multivariable hazard ratios of 0.69 (0.47–1.01) (P for trend = 0.05). Similar but nonsignificant inverse associations were observed for α-, γ-, and δ-tocopherols but not for β-tocopherol. These results were similar regardless of the presence of a history of stroke. Dietary tocopherol intake was inversely associated with risk of disabling dementia, but its independent effect was uncertain owing to a high intercorrelation of α-linolenic linoleic acids with total tocopherol intake. Even with such confounding, a diet high in tocopherols may help prevent the onset of dementia.

## Introduction

Vitamin E exists in four main forms, i.e., α-, β-, γ-, and δ-tocopherols, and is abundant in edible plant fat and fish. It has been reported that vitamin E reduces reactive oxygen species^1^ and, in the human body, may prevent oxidized nucleic acids that could cause neurodegenerative diseases such as disabling dementia^2^. Therefore, tocopherols are expected to have clear antiatherosclerotic and neuronal protective functions that contribute to the prevention of such diseases. However, evidence from epidemiological studies in Europe and the United States has been controversial^3–5^, and no prospective studies have been reported in Asian populations.

We therefore investigated whether dietary tocopherol intake is associated with reduced risk of dementia in a community-based cohort study of Japanese men and women, whose mean intake of dietary tocopherols was lower than the intake reported to be adequate among men and women in Japanese communities^6^.

## Participants and methods

The Circulatory Risk in Communities Study (CIRCS) is an ongoing dynamic community-based prospective study involving five communities in Japan. Details of the CIRCS protocol have been described elsewhere^7^. In the present study, we included three communities, Ikawa (a rural community of Akita Prefecture in northeastern Japan), Kyowa (a rural community of Ibaraki Prefecture in mid-eastern Japan), and Minami-Takayasu (a suburb of Osaka Prefecture in mid-western Japan), where disabling dementia surveillance is being conducted.

We set the risk set as 3750 people aged 40–64 years living in these three communities who participated in an annual cardiovascular risk survey from 1985 to 1999. They were followed up to confirm incident dementia from 1999 through 2020 (except for from April 2005 to April 2008, for which period the data were unavailable) in Kyowa; from 1999 to 2019 in Ikawa; and from 2006 to 2019 in Yao. The participants were primarily healthy at the time of the dietary surveys and we did not ask about a history of dementia in the surveys. Instead, we excluded persons who participated in the dietary survey at least 5 years before receiving a dementia diagnosis (n = 2 excluded) to minimize the possibility of reverse causation. We further excluded nine persons with invalid dietary data. In the end, 3739 participants were used for the analysis.

The intake of energy and nutrients from each meal was calculated on the basis of the Standard Tables of Food Composition in Japan, 2015 (7th revised edition)^8^. All nutrient variables were adjusted for energy intake using the nutrient residual model.

Diagnoses of disabling dementia were performed by attending physicians under the National Long-Term Care Insurance System, which is a compulsory insurance for all individuals aged 40 years or older in Japan^9^. To apply for care under this system, the individual’s attending physician is required to evaluate the individual’s activities of daily living in relation to dementia. The criteria of disabling dementia were the same as those of our previous study (certified for disability under the long-term-care insurance program and the grade of activities of daily living related to dementia of ≥ IIa)^10^. The validation of these criteria was previously validated against diagnoses by certified psychiatrists with high specificity (96%) and moderate sensitivity (73%)^11^.

Potential risk factors for disabling dementia were measured at the baseline examination^7^. In brief, we measured the arterial systolic and fifth-phase diastolic blood pressures using standard mercury sphygmomanometers on the right arm of the participants, who were quietly seated after having rested for at least 5 min. When the first systolic blood pressure reading was ≥ 140 mmHg and/or the diastolic blood pressure was ≥ 90 mmHg, the  blood pressure measurement was repeated. In this case, the second reading was used in the analysis; otherwise, was so the first reading. Height without shoes and weight in light clothing were measured, and body mass index was calculated as weight (kg) divided by square of height (m^2^). Face-to-face interviews were conducted to determine drinking (noncurrent or current) and smoking (never, ex, or current) statuses, and use of antihypertensive medication, cholesterol-lowering medication, and diabetes medication. Serum glucose and total cholesterol were measured at baseline without a fasting requirement. Diabetes mellitus was defined as fasting serum glucose ≥ 126 mg/dL or non-fasting serum glucose ≥ 200 mg/dL or being under medication for diabetes. In the present study, 90% of stroke occurrence was confirmed on the basis of computed tomography or magnetic resonance imaging using standardized criteria^12^, whilst the rest were determined using previously reported clinical criteria for diagnosis without imaging^13,14^. Stroke was defined as a rapid-onset focal neurological disorder persisting for ≥ 24 h or until death.

The age- and sex-adjusted means and proportions of the characteristics of the study participants at the time of the dietary surveys were compared according to the quartiles of intakes of total, α-, β-, γ-, and δ-tocopherols by means of analyses of covariance. We conducted proportional hazards regression analysis using SAS 9.4 (SAS Institute, Cary, NC, USA). In model 1, we calculated age- and sex-adjusted and area-stratified hazard ratios and 95% confidence intervals for disabling dementia. We further adjusted for smoking status (never, former, current 1–20 or ≥ 20 cigarettes/day), intake of energy (continuous) and docosahexaenoic acid (DHA) + docosahexaenoic acid (EPA) (ω-3 polyunsaturated fatty acid of marine origin) in model 2. Because of the high intercorrelations between total tocopherols and α-linolenic acid (Pearson correlation coefficient = 0.93), and linoleic acid (Pearson correlation coefficient = 0.92), we further adjusted for α-linolenic acid in model 3 and for linoleic acid in model 4. To distinguish between different types of dementia, disabling dementia cases were classified into two categories: with a history of stroke and without a history of stroke, following the systematic stroke registration described elsewhere^15^ as well as self-reports in the dietary surveys. The end of the follow-up period was set for the end of 2015 for Kyowa and for the end of 2018 for Ikawa and Yao, considering the availability of the stroke registry data. All probability values for the statistical tests were 2-tailed, and values below 0.05 were considered significant.

Informed consent was obtained from community leaders. Individual consent was not required for the analysis of this study, since it was conducted as a secondary use of data obtained for public health practice on cardiovascular disease prevention in the local community at that time. Adhering to relevant guidelines and regulations afterwards, participants were retrospectively given the opportunity to withdraw their data from analysis, and the consent was considered to have been obtained if the participant did not decline in this study.

This study was approved by the institutional review boards of both the Osaka Center for Cancer and Cardiovascular Disease Prevention and the University of Tsukuba.

### Ethics approval

This study was approved by the institutional review boards of both the Osaka Center for Cancer and Cardiovascular Disease Prevention (approval number: R2-Rinri-4) and of the University of Tsukuba (approval number: 66-8).

### Consent to participate

Informed consent was obtained from community leaders. Individual consent was not required for the analysis of this study, since it was conducted as a secondary use of data obtained for public health practice on cardiovascular disease prevention in the local community at that time. Adhering to relevant guidelines and regulations afterwards, participants were retrospectively given the opportunity to withdraw their data from analysis, and the consent was considered to have been obtained if the participant did not decline in this study.

### Consent for publication

All the listed authors have approved the manuscript before submission.

## Results

At baseline, the mean value of carbohydrate was inversely correlated, and the proportion of women and the mean values of total fat, total fiber, vegetables, sodium, α-linolenic acids, and linoleic acid were positively correlated with all of the tocopherol constituents (Table 1, Supplemental Tables 1–4).

**Table 1:** Characteristics of participants aged 40–64 years, according to quartiles of total tocopherol intake at dietary surveys, CIRCS 1985–1999.

	Total tocopherol				
	Q1 (Low)	Q2	Q3	Q4 (High)	P for trend
Median vitamin E^a^ (mg/day)	11.9	18.4	24.2	33.3	
Age (years)	51.9	51.3	51.1	50.7	< 0.0001
Women (%)	39.2	51.3	61.9	71.0	< 0.0001
Body mass index (kg/m^2^)	23.7	23.8	23.7	23.8	0.74
Current smoker (%)	37.7	28.7	23.4	19.5	0.28
Current drinker (%)	52.0	40.6	35.7	29.0	0.21
Systolic blood pressure (mmHg)	132	131	131	129	0.07
Diastolic blood pressure (mmHg)	82.4	81.7	80.8	80.2	0.18
Antihypertensive medication (%)	13.0	11.1	12.8	12.6	0.51
Diabetes mellitus (%)	5.3	5.0	4.9	3.8	0.87
History of stroke (%)	1.2	1.0	0.7	1.2	0.61
Serum total cholesterol (mg/dL)	199	201	199	202	0.21
Cholesterol-lowering medication (%)	1.7	2.1	2.5	2.4	0.73
Energy (kcal/day)	2011	1955	1966	1973	< 0.0001
Carbohydrate (g/day)	311	288	281	269	< 0.0001
Total fat (g/day)	35.5	35.5	43.6	49.6	< 0.0001
Protein (g/day)	67.4	70.2	72.2	71.8	< 0.0001
Total fiber (g/day)	12.6	14.2	15.4	17.4	< 0.0001
Vegetables (g/day)	227	258	296	322	< 0.0001
Fruit (g/day)	132	142	144	160	0.33
Fish (g/day)	98.9	100.1	98.8	88.5	0.08
Meats (g/day)	46.7	49.5	50.4	48.0	0.12
Sodium (mg/day)	4308	4528	4728	4774	< 0.0001
DHA + EPA (mg/day)	991	979	968	806	0.03
α-Linolenic acid (mg/day)	686	1139	1534	2354	< 0.0001
Linoleic acid (mg/day)	4932	7390	9553	13,762	< 0.0001

^a^Energy-adjusted values by nutrient residual model.

As shown in Table 2, we observed an inverse association between dietary intakes of total tocopherol with risk of disabling dementia. The multivariable hazard ratios and 95% confidence intervals for persons with the second, third, and highest quartiles of total tocopherol in model 2 were 1.01 (0.82–1.25), 0.95 (0.77–1.18), and 0.79 (0.63–1.00), respectively, as compared with the lowest quartile (P for trend = 0.05). Similar but nonsignificant associations with disabling dementia were observed for α-, γ-, and δ-tocopherols. No association was observed for β-tocopherol.

**Table 2:** Multivariable hazard ratios and 95% confidence intervals of disability dementia according to quartiles of total tocopherol, α-, β-, γ- and δ-tocopherol intakes.

	Q1	Q2	Q3	Q4	Trend P
	**Total tocopherol**				
Person-years	14,363	14,974	14,980	15,365	
Number of cases	178	179	168	145	
Model 1	1.00	0.99 (0.81–1.22)	0.93 (0.75–1.16)	0.79 (0.63–1.00)	0.04
Model 2	1.00	1.01 (0.82–1.25)	0.95 (0.77–1.18)	0.79 (0.63–1.00)	0.05
Model 3	1.00	0.89 (0.71–1.12)	0.74 (0.56–0.98)	0.50 (0.34–0.74)	0.001
Model 4	1.00	0.97 (0.78–1.22)	0.88 (0.67–1.16)	0.69 (0.47–1.01)	0.05
	**α-Tocopherol**				
Person-years	14,480	15,011	15,023	15,167	
Number of cases	177	148	175	170	
Model 1	1.00	0.73 (0.59–0.91)	0.89 (0.72–1.10)	0.79 (0.64–0.99)	0.16
Model 2	1.00	0.74 (0.59–0.93)	0.91 (0.74–1.14)	0.81 (0.65–1.02)	0.19
Model 3	1.00	0.74 (0.59–0.92)	0.90 (0.72–1.13)	0.80 (0.62–1.03)	0.25
Model 4	1.00	0.75 (0.60–0.94)	0.94 (0.66–1.18)	0.85 (0.66–1.09)	0.53
	**β-Tocopherol**				
Person-years	14,742	15,118	14,865	14,957	
Number of cases	187	172	161	150	
Model 1	1.00	1.03 (0.84–1.27)	0.91 (0.73–1.13)	0.94 (0.76–1.17)	0.39
Model 2	1.00	1.04 (0.84–1.28)	0.92 (0.74–1.15)	0.93 (0.74–1.16)	0.48
Model 3	1.00	1.02 (0.82–1.27)	0.90 (0.70–1.14)	0.88 (0.66–1.18)	0.28
Model 4	1.00	1.07 (0.86–1.33)	0.97 (0.76–1.24)	1.00 (0.75–1.34)	0.85
	**γ-Tocopherol**				
Person-years	14,493	14,972	14,884	15,333	
Number of cases	186	163	174	147	
Model 1	1.00	0.91 (0.74–1.12)	0.97 (0.78–1.19)	0.87 (0.70–1.09)	0.34
Model 2	1.00	0.92 (0.74–1.14)	0.97 (0.79–1.20)	0.86 (0.68–1.08)	0.39
Model 3	1.00	0.85 (0.67–1.08)	0.83 (0.63–1.11)	0.64 (0.42–0.99)	0.06
Model 4	1.00	0.95 (0.75–1.20)	1.03 (0.77–1.38)	0.95 (0.62–1.46)	0.92
	**δ-Tocopherol**				
Person-years	14,572	14,740	15,210	15,160	
Number of cases	175	161	169	165	
Model 1	1.00	0.93 (0.75–1.15)	0.90 (0.73–1.11)	0.86 (0.69–1.07)	0.16
Model 2	1.00	0.92 (0.74–1.13)	0.90 (0.73–1.11)	0.83 (0.67–1.04)	0.14
Model 3	1.00	0.88 (0.70–1.10)	0.83 (0.66–1.06)	0.73 (0.55–0.99)	0.04
Model 4	1.00	0.92 (0.74–1.15)	0.91 (0.72–1.15)	0.85 (0.64–1.14)	0.28

Model 1: Adjusted for age and sex, and stratified by area.

Model 2: Multivariable model adjusted further for energy, smoking status, and DHA + EPA.

Model 3: Multivariable model adjusted further for variables in model 2 and α-linolenic acid.

Model 4: Multivariable model adjusted further for variables in model 2 and linoleic acid.

After further adjustment for linoleic acid, we still found an inverse association of dietary intake of total tocopherols with risk of incident dementia (model 4). The multivariable hazard ratios and 95% confidence intervals for persons with the second, third, and highest quartiles of total tocopherol were 0.97 (0.78–1.22), 0.88 (0.67–1.16), and 0.69 (0.47–1.01), respectively, as compared with the lowest quartiles (P for trend = 0.05). Adjustment for α-linolenic acid strengthened these associations (model 3), whilst adjustment for linoleic acid attenuated the associations (model 4). It is noteworthy that the multivariable hazard ratios and 95% confidence intervals for the respective quartiles of α-linolenic acid in model 2 were 1.05 (0.85–1.29), 0.93 (0.75–1.15), and 0.89 (0.71–1.13), respectively, as compared with the lowest quartile (P for trend = 0.21) and those for the respective quartiles of linoleic acid in model 2 were 0.95 (0.77–1.17), 0.86 (0.69–1.07), and 0.90 (0.72–1.13), respectively, as compared with the lowest quartile (P for trend = 0.26) (data not shown).

These associations between dietary tocopherol intakes and disabling dementia were similarly observed when we analyzed the data for the participants with or without a history of stroke (Table 3).

**Table 3:** Multivariable hazard ratios and 95% confidence intervals of disability dementia according to quartiles of total tocopherol, α-, β-, γ- and δ-tocopherol intakes.

	Q1	Q2	Q3	Q4	Trend P
	**Total tocopherol**				
Person-years	14,363	14,974	14,980	15,365	
Dementia with a history of stroke					
Number of cases	41	33	21	18	
Model 1	1.00	0.89 (0.56–1.40)	0.62 (0.36–1.06)	0.60 (0.34–1.06)	0.04
Model 2	1.00	0.88 (0.56–1.40)	0.63 (0.37–1.07)	0.60 (0.33–1.06)	0.04
Dementia without a history of stroke					
Number of cases	109	109	111	88	
Model 1	1.00	0.95 (0.73–1.24)	0.96 (0.73–1.26)	0.77 (0.57–1.03)	0.09
Model 2	1.00	0.97 (0.74–1.27)	0.98 (0.74–1.28)	0.77 (0.57–1.03)	0.09
	**α-Tocopherol**				
Person-years	14,480	15,011	15,023	15,167	
Dementia with a history of stroke					
Number of cases	30	33	28	22	
Model 1	1.00	1.09 (0.66–1.80)	1.03 (0.61–1.74)	0.84 (0.47–1.48)	0.52
Model 2	1.00	1.11 (0.67–1.84)	1.03 (0.61–1.76)	0.83 (0.46–1.50)	0.51
Dementia without a history of stroke					
Number of cases	112	83	110	112	
Model 1	1.00	0.60 (0.45–0.80)	0.80 (0.61–1.05)	0.73 (0.55–0.96)	0.15
Model 2	1.00	0.61 (0.45–0.81)	0.82 (0.63–1.08)	0.75 (0.57–1.00)	0.22
	**β-Tocopherol**				
Person-years	14,742	15,118	14,865	14,957	
Dementia with a history of stroke					
Number of cases	41	28	24	20	
Model 1	1.00	0.82 (0.51–1.34)	0.77 (0.46–1.29)	0.72 (0.42–1.25)	0.24
Model 2	1.00	0.83 (0.51–1.35)	0.80 (0.48–1.34)	0.73 (0.41–1.28)	0.27
Dementia without a history of stroke					
Number of cases	116	106	103	92	
Model 1	1.00	1.05 (0.80–1.36)	0.92 (0.70–1.20)	0.93 (0.70–1.23)	0.45
Model 2	1.00	1.05 (0.80–1.37)	0.93 (0.70–1.22)	0.91 (0.68–1.21)	0.37
	**γ-Tocopherol**				
Person-years	14,493	14,972	14,884	15,333	
Dementia with a history of stroke					
Number of cases	41	24	30	18	
Model 1	1.00	0.68 (0.41–1.13)	0.94 (0.58–1.52)	0.67 (0.38–1.19)	0.34
Model 2	1.00	0.68 (0.41–1.14)	0.95 (0.58–1.54)	0.67 (0.37–1.20)	0.36
Dementia without a history of stroke					
Number of cases	116	107	107	87	
Model 1	1.00	0.94 (0.72–1.23)	0.95 (0.73–1.25)	0.85 (0.64–1.14)	0.31
Model 2	1.00	0.95 (0.72–1.24)	0.95 (0.72–1.25)	0.83 (0.62–1.12)	0.25
	**δ-Tocopherol**				
Person-years	14,572	14,740	15,210	15,160	
Dementia with a history of stroke					
Number of cases	41	24	22	26	
Model 1	1.00	0.64 (0.38–1.06)	0.58 (0.35–0.98)	0.75 (0.45–1.24)	0.24
Model 2	1.00	0.63 (0.38–1.04)	0.57 (0.34–0.97)	0.74 (0.44–1.22)	0.23
Dementia without a history of stroke					
Number of cases	108	107	110	92	
Model 1	1.00	0.96 (0.74–1.26)	0.95 (0.73–1.24)	0.76 (0.57–1.01)	0.07
Model 2	1.00	0.95 (0.72–1.24)	0.94 (0.72–1.23)	0.73 (0.55–0.98)	0.04

Model 1: Adjusted for age and sex, and stratified by area.

Model 2: Multivariable model adjusted further for energy, smoking status, and DHA + EPA.

Because of the lack of information regarding registration of dementia between 2005 and 2008 in Kyowa, we performed a sensitivity analysis starting from the follow-up from 2009. The associations did not change essentially (Supplemental Table 5).

## Discussion

We found an inverse association between dietary tocopherol intake and risk of incident disabling dementia in the Japanese population. Since tocopherols, α-linolenic acid, and linoleic acid are often found together in food sources (mainly vegetable oils), dietary intake of tocopherol was highly correlated with intakes of α-linolenic acid and linoleic acid, and the observed tocopherol–dementia associations were changed after adjustment for α-linolenic and linoleic acids.

Some animal and human studies support our findings that vitamin E is useful in preventing dementia. An antioxidant effect of α-tocopherol was reported in a murine liver model where rats fed with supplemental vitamin E had higher liver α-tocopherol levels and decreased lipid peroxidation after the introduction of an oxidizing agent^1^. Beyond antioxidant effects, γ-tocopherol increased sodium excretion in a dose-dependent manner in rats fed an NaCl-rich diet^16^, suggesting that dietary γ-tocopherol may prevent vascular dementia associated with hypertension. Furthermore, triple transgenic mice for Alzheimer disease showed lower natural killer cell activity than that of the controls before the onset of Alzheimer disease^17^, whilst dietary supplementation of vitamin E is known to enhance natural killer cell proliferation in older adults^18^.

Dietary studies in humans also reported generally similar results. The Rotterdam Study of 5395 participants, aged 55 years or older and with a mean follow-up of 9.6 years, showed that participants in the highest tertile of dietary vitamin E had a lower risk of developing dementia than did those in the lowest tertile (HR, 0.76; 95% CI 0.60–0.96)^5^. As for Alzheimer disease, a meta-analysis that enrolled 904 dementia patients and 1153 controls in Europe reported that Alzheimer disease patients had lower concentrations of serum vitamin E than did the healthy controls (weighted mean difference =  − 6.811 μmol/L, 95% CI − 8.998 to − 4.625, P < 0.001)^19^. Another prospective study of 815 US residents aged 65 years or older, free of dementia at baseline, and with a median follow-up of 3.9 years showed that dietary vitamin E intake was inversely associated with risk of Alzheimer disease only among persons who were *APOE* ε4 noncarriers. In that study, the multivariable relative risks and 95% confidence intervals for the second, third, fourth, and highest quartiles of vitamin E intake were 0.63 (0.20–2.01), 0.34 (0.13–0.89), 0.23 (0.08–0.67), and 0.17 (0.06–0.47), respectively, as compared with the lowest quartile^3^. Although we did not have *APOE* information for our participants, the reported ε4 allele frequency in Japanese individuals (11.6%) is not high when compared with that in white individuals (24.0%)^20^, and thus, this *APOE* genotype is unlikely to have largely impacted our results. No prospective study has been conducted to show the association between dietary tocopherol intake and risk of vascular dementia.

In contrast, previous studies did not show any associations between dietary vitamin E and risk of dementia. A study of 980 participants aged 65 years or older with a mean follow-up of 4 years, in the Washington Heights-Inwood Columbia Aging Project, showed weaker and nonsignificant associations between dietary vitamin E intake and decreased risk of Alzheimer disease (P for trend = 0.83)^4^. The other studies reported that use of supplemental vitamin E was not associated with a decreased risk of Alzheimer disease^21,22^. As for randomized controlled studies, a study of 3786 healthy men aged 60 years or older showed that vitamin E supplementation did not prevent dementia: the yielded hazard ratio was 0.88 (95% CI 0.64–1.20) as compared with the placebo^23^. This discrepancy might be due to some factors that may affect the bioavailability of vitamin E; for example, it has been reported that age, sex, obesity, smoking, low-density lipoprotein cholesterol, high-density lipoprotein cholesterol, triglycerides, and fasting blood glucose affect serum vitamin E concentrations^24^. Differences in the follow-up periods may also have influenced each result.

The effect of ω-3 polyunsaturated fatty acids and ω-6 polyunsaturated fatty acids, which may be beneficial in the prevention of dementia, should be taken into account because tocopherols, ω-3 polyunsaturated fatty acids, and ω-6 polyunsaturated fatty acids, especially α-linolenic acid and linoleic acid, are found in shared food sources. In the CIRCS, we previously reported that serum α-linolenic acid, but not EPA or DHA, was inversely associated with risk of disabling dementia^25^. In the present study, we observed that the association between intakes of all tocopherol constituents and risk of disabling dementia were strengthened when we adjusted for dietary α-linolenic acid intake. Since edible plant fat and fish abundantly contain both tocopherol and α-linolenic acid, the strong intercorrelation (r = 0.93) dictates that the individual impacts of these nutrients cannot be completely distinguished in a real-world observational study. Similarly, studies reported that simultaneous intake of tocopherol, α-linolenic and linoleic acid from foods and dietary patterns were associated with lowered risk of dementia. A 3.9-year cohort study of 2148 persons aged 65 years or older showed that dietary patterns characterized by a diet rich in ω-3 PUFA, ω-6 PUFA, vitamin E, and folate were significantly associated with a lower risk of incident Alzheimer disease (P for trend = 0.01)^26^.

The strength of the present study involved the use of a community-based sample of relatively young individuals (aged 59.7 years on average) and a long follow-up (24.1 years on average). Our findings are thus likely to be applicable to other Asian populations. Furthermore, we estimated intakes of α, β, γ, and δ-tocopherols and evaluated the associations between intake of each of these tocopherols and risk of disabling dementia.

Several limitations of this study should be noted. First, attending physicians diagnosed disabling dementia, but the criteria for such diagnoses were validated against diagnosis by certified psychiatrists in a previous study^11^. The sensitivity and specificity for these diagnoses were 73% and 96%. Second, we analyzed dietary survey data using 24-h recall surveys, which may be insufficient to reflect typical diets for individuals; we previously performed a reproducibility study of 232 Japanese residents aged 40–69 years and reported that the correlation coefficients of total tocopherols examined a year apart in the same season throughout 1973–1999 was moderate (0.36 for both men and women)^27^. In the present study period (1984–2000), the correlation coefficients between the tocopherol values examined a year apart were moderate to high except for those of γ-tocopherol (r = 0.44 for total tocopherol, r = 0.76 for α-tocopherol, r = 0.72 for β-tocopherol, r = 0.25 for γ-tocopherol, and r = 0.80 for δ-tocopherol). Third, we did not have data on the type of dementia (i.e., Alzheimer or vascular dementia), but we did have information on dementia with or without a history of stroke. Although the number of cases was relatively small, the association of dietary tocopherols intake was similarly observed with disabling dementia regardless of the presence of a history of stroke (Table 3). Finally, since we did not survey prevalent dementia at baseline, we set the baseline at least 5 years before the beginning of the dementia survey (median follow-up = 19.7 years) to reduce the possibility of reverse causation. In addition, we did not have the information on the dementia registry in Kyowa between 2005 and 2008. We performed sensitivity analyses following up all participants from 2009 and found no change in the results.

In conclusion, dietary tocopherol intake was inversely associated with risk of disabling dementia among Japanese, suggesting that persons with a diet consisting of sufficient tocopherol intake are less likely to develop disabling dementia. However, it is difficult to separate the effect of tocopherol intake from that of α-linolenic acid owing to the strong correlation with dietary tocopherol intake. Even with such confounding, a diet high in tocopherols may help prevent the onset of dementia.

## Supplementary Information

Supplementary Tables.

## Data Availability

The datasets generated during and/or analyzed during the current study are not publicly available owing to the restriction policy of the CIRCS committee but are available from the corresponding author upon reasonable request and with permission of the CIRCS committee.

## Code availability

The code for the study analyses is available from the corresponding author upon reasonable request and with permission of the CIRCS committee.
