# Supplementary material for: Dietary intake of tocopherols and risk of incident disabling dementia
Source: Sci Rep. 2021 Aug 12;11:16429. doi: 10.1038/s41598-021-95671-7 (PMC8361201; doi:10.1038/s41598-021-95671-7)
Supplement: Supplementary file 1 — Supplementary Tables. [file 41598_2021_95671_MOESM1_ESM.pdf]

TITLE: Dietary intake of tocopherols and risk of incident disabling dementia

Shoko Aoki[1], Kazumasa Yamagishi, MD, PhD[1,2,3], Koutatsu Maruyama, PhD[4], Rie Kishida, PhD[1], Ai Ikeda, PhD[5], Mitsumasa Umesawa, MD, PhD[1,6], Cui Renzhe, MD, PhD[7], Yasuhiko Kubota, MD, MPH, PhD[2], Mina Hayama-Terada, MD, PhD[2,8], Yuji Shimizu, MD, PhD[2], Isao Muraki, MD, PhD[7], Hironori Imano, MD, PhD[7], Tomoko Sankai, MD, PhD[9], Takeo Okada, MD, PhD[2], Akihiko Kitamura, MD, PhD[2,10], Masahiko Kiyama, MD, PhD[2], Hiroyasu Iso, MD, MPH, PhD[1,7]

Supplemental Table 1. Characteristics of participants aged 40–64 years, according to quartiles of α-tocopherol intake at the time of the dietary surveys, CIRCS 1985–1999

	α-tocopherol				
	Q1 (Low)	Q2	Q3	Q4 (High)	P for trend
Median α-tocopherol[a], mg/day	4.1	6.1	7.8	10.8	
Age, y	51.3	51.3	51.0	51.4	0.49
Women, %	39.6	51.3	63.0	69.5	<.0001
Body mass index, kg/m^2	23.7	23.8	23.7	23.9	0.68
Current smoker, %	37.5	28.5	23.6	19.8	0.37
Current drinker, %	48.1	43.7	35.7	29.8	0.12
Systolic blood pressure, mmHg	133	130	129	130	0.00
Diastolic blood pressure, mmHg	82.5	81.3	80.7	80.6	0.21
Antihypertensive medication, %	14.2	12.1	9.4	13.8	0.01
Diabetes mellitus, %	5.9	4.9	3.8	4.5	0.75
History of stroke, %	1.1	1.1	1.3	0.6	0.59
Serum total cholesterol, mg/dL	198	200	201	201	1.00
Cholesterol-lowering medication, %	1.7	2.6	2.7	1.7	0.26
Energy, kcal/day	1971	2001	1967	1966	<.0001
Carbohydrate, g/day	306	291	281	271	0.01
Total fat, g/day	36.7	36.7	46.5	50.1	<.0001
Protein, g/day	63.8	70.4	72.1	75.3	<.0001
Total fiber, g/day	12.5	14.0	15.7	17.3	<.0001
Vegetables, g/day	204	248	294	358	<.0001
Fruit, g/day	110	141	157	171	<.0001
Fish, g/day	75.4	91.4	101.6	117.8	<.0001
Meats, g/day	47.0	54.2	50.5	42.8	0.0003
Sodium, mg/day	4297	4568	4727	4746	<.0001
DHA+EPA, mg/day	642	939	978	1183	0.0003
α-linolenic acid, mg/day	947	1288	1549	1930	<.0001
Linoleic acid, mg/day	6590	8307	9551	11191	<.0001

[a]Energy-adjusted values by nutrient residual model.

Supplemental Table 2. Characteristics of participants aged 40–64 years, according to quartiles of β-tocopherol intake at dietary surveys, CIRCS 1985–1999

	β-tocopherol				
	Q1 (Low)	Q2	Q3	Q4 (High)	P for trend
Median β-tocopherol[a], mg/day	0.2	0.3	0.4	0.6	
Age, y	52.4	51.2	50.9	50.5	<.0001
Women, %	43.9	54.0	59.7	65.9	<.0001
Body mass index, kg/m^2	23.7	23.7	23.6	24.0	0.09
Current smoker, %	35.5	27.0	25.5	21.4	0.13
Current drinker, %	47.6	40.3	36.7	32.7	0.95
Systolic blood pressure, mmHg	132	130	130	130	0.49
Diastolic blood pressure, mmHg	81.8	81.3	80.9	81.1	0.88
Antihypertensive medication, %	12.6	12.7	10.8	13.4	0.15
Diabetes mellitus, %	5.4	5.2	4.7	3.8	0.89
History of stroke, %	1.0	1.0	0.7	1.4	0.25
Serum total cholesterol, mg/dL	198	201	201	200	0.27
Cholesterol-lowering medication, %	2.0	1.7	1.7	3.2	0.06
Energy, kcal/day	1987	1962	1972	1983	0.0002
Carbohydrate, g/day	303	288	281	278	0.01
Total fat, g/day	36.9	36.9	45.0	50.6	<.0001
Protein, g/day	69.9	70.4	71.3	70.0	0.009
Total fiber, g/day	13.3	14.3	15.3	16.5	<.0001
Vegetables, g/day	262	277	285	280	0.03
Fruit, g/day	147	142	148	141	0.27
Fish, g/day	116.1	102.4	90.4	77.5	<.0001
Meats, g/day	46.6	50.2	52.3	45.5	<.0001
Sodium, mg/day	4329	4412	4649	4949	<.0001
DHA+EPA, mg/day	1186	997	899	661	<.0001
α-linolenic acid, mg/day	808	1240	1566	2099	<.0001
Linoleic acid, mg/day	5250	7824	9834	12,729	<.0001

[a]Energy-adjusted values by nutrient residual model.

Supplemental Table 3. Characteristics of participants aged 40–64 years, according to quartiles of γ-tocopherol intake at dietary surveys, CIRCS 1985–1999

	γ-tocopherol				
	Q1 (Low)	Q2	Q3	Q4 (High)	P for trend
Median γ-tocopherol[a], mg/day	4.7	8.7	12.4	18.4	
Age, y	52.2	51.1	51.3	50.4	<.0001
Women, %	41.5	52.6	59.8	69.5	<.0001
Body mass index, kg/m^2	23.7	23.8	23.7	23.8	0.50
Current smoker, %	35.3	28.7	24.8	20.6	0.87
Current drinker, %	51.7	39.4	35.8	30.4	0.01
Systolic blood pressure, mmHg	132	130	131	129	0.03
Diastolic blood pressure, mmHg	82.6	81.3	81.2	79.9	0.02
Antihypertensive medication, %	13.0	12.7	11.3	12.5	0.47
Diabetes mellitus, %	5.4	4.8	4.5	4.4	0.92
History of stroke, %	1.0	1.2	0.7	1.2	0.40
Serum total cholesterol, mg/dL	199	200	200	201	0.95
Cholesterol-lowering medication, %	1.7	2.1	2.1	2.7	0.44
Energy, kcal/day	1992	1993	1949	1971	<.0001
Carbohydrate, g/day	307	295	279	268	<.0001
Total fat, g/day	35.3	35.3	43.8	49.6	<.0001
Protein, g/day	69.3	72.1	70.0	70.1	<.0001
Total fiber, g/day	13.2	14.6	15.0	16.7	<.0001
Vegetables, g/day	245	270	281	306	<.0001
Fruit, g/day	139	144	147	148	0.98
Fish, g/day	109.4	104.8	90.3	81.9	<.0001
Meats, g/day	44.7	48.7	52.3	48.9	0.004
Sodium, mg/day	4378	4676	4570	4714	<.0001
DHA+EPA, mg/day	1126	1024	889	705	<.0001
α-linolenic acid, mg/day	666	1129	1536	2382	<.0001
Linoleic acid, mg/day	4674	7367	9642	13,955	<.0001

[a]Energy-adjusted values by nutrient residual model.

Supplemental Table 4. Characteristics of participants aged 40–64 years, according to quartiles of δ-tocopherol intake at dietary surveys, CIRCS 1985–1999

	δ-tocopherol				
	Q1 (Low)	Q2	Q3	Q4 (High)	P for trend
Median δ-tocopherol[a], mg/day	1.4	2.6	3.6	5.3	
Age, y	51.3	51.1	51.3	51.4	0.86
Women, %	43.9	53.4	59.3	67.0	<.0001
Body mass index, kg/m^2	23.7	23.7	23.7	24.0	0.20
Current smoker, %	33.5	28.7	26.2	20.9	0.89
Current drinker, %	49.7	40.2	37.3	30.1	0.09
Systolic blood pressure, mmHg	131	131	130	130	0.54
Diastolic blood pressure, mmHg	82.3	81.3	80.5	81.0	0.12
Antihypertensive medication, %	11.6	12.5	11.1	14.3	0.17
Diabetes mellitus, %	5.8	3.6	5.3	4.4	0.18
History of stroke, %	1.0	0.6	1.0	1.5	0.18
Serum total cholesterol, mg/dL	199	201	200	201	0.70
Cholesterol-lowering medication, %	0.9	3.2	1.6	3.0	0.001
Energy, kcal/day	1989	1997	1970	1949	0.001
Carbohydrate, g/day	299	293	285	273	0.04
Total fat, g/day	39.0	39.0	46.2	49.5	<.0001
Protein, g/day	68.7	70.7	71.0	71.1	<.0001
Total fiber, g/day	12.8	14.3	15.3	17.1	<.0001
Vegetables, g/day	242	276	290	296	<.0001
Fruit, g/day	145	146	138	149	0.31
Fish, g/day	107.9	102.7	93.2	82.5	<.0001
Meats, g/day	48.7	51.9	50.7	43.2	0.02
Sodium, mg/day	4226	4519	4634	4958	<.0001
DHA+EPA, mg/day	1102	1008	910	723	<.0001
α-linolenic acid, mg/day	764	1227	1571	2152	<.0001
Linoleic acid, mg/day	5361	7894	9639	12,744	<.0001

[a]Energy-adjusted values by nutrient residual model.

Supplemental Table 5. Multivariable hazard ratios and 95% confidence intervals of disability dementia in follow-up of all participants from 2009 according to quartiles of total tocopherol, α-, β-, γ- and δ-tocopherol intakes

	Q1	Q2		Q3		Q4		Trend P
**Total tocopherol**								
	Q1	Q2		Q3		Q4		Trend P
Person-years	13,873	14,458		14,571		15,016		
Number of cases	156	152		140		127		
Model 1	1.00	0.97	(0.77 - 1.21)	0.89	(0.71 - 1.12)	0.79	(0.62 - 1.00)	0.04
Model 2	1.00	0.99	(0.79 - 1.23)	0.91	(0.72 - 1.15)	0.78	(0.61 - 1.00)	0.04
Model 3	1.00	0.83	(0.65 - 1.06)	0.66	(0.49 - 0.89)	0.43	(0.28 - 0.66)	<.0001
Model 4	1.00	0.94	(0.74 - 1.20)	0.83	(0.62 - 1.11)	0.67	(0.44 - 1.01)	0.05
**α-tocopherol**								
	Q1	Q2		Q3		Q4		Trend P
Person-years	13,966	14,562		14,675		14,714		
Number of cases	158	129		146		142		
Model 1	1.00	0.72	(0.57 - 0.91)	0.84	(0.67 - 1.06)	0.75	(0.59 - 0.95)	0.07
Model 2	1.00	0.72	(0.57 - 0.91)	0.86	(0.68 - 1.09)	0.76	(0.59 - 0.97)	0.10
Model 3	1.00	0.70	(0.55 - 0.89)	0.82	(0.64 - 1.05)	0.70	(0.53 - 0.93)	0.05
Model 4	1.00	0.73	(0.57 - 0.92)	0.88	(0.66 - 1.12)	0.78	(0.59 - 1.02)	0.20
**β-tocopherol**								
	Q1	Q2		Q3		Q4		Trend P
Person-years	14,277	14,610		14,460		14,570		
Number of cases	161	148		133		133		
Model 1	1.00	1.02	(0.82 - 1.28)	0.88	(0.69 - 1.11)	0.96	(0.76 - 1.22)	0.48
Model 2	1.00	1.04	(0.83 - 1.30)	0.90	(0.71 - 1.13)	0.95	(0.75 - 1.21)	0.45
Model 3	1.00	1.01	(0.80 - 1.28)	0.85	(0.66 - 1.11)	0.88	(0.65 - 1.21)	0.29
Model 4	1.00	1.08	(0.85 - 1.37)	0.95	(0.73 - 1.24)	1.05	(0.77 - 1.43)	0.97
**γ-tocopherol**								
	Q1	Q2		Q3		Q4		Trend P
Person-years	13,984	14,493		14,446		14,994		
Number of cases	158	146		141		130		
Model 1	1.00	0.96	(0.76 - 1.20)	0.92	(0.73 - 1.16)	0.89	(0.70 - 1.14)	0.33
Model 2	1.00	0.97	(0.77 - 1.22)	0.93	(0.74 - 1.18)	0.88	(0.69 - 1.13)	0.29
Model 3	1.00	0.87	(0.68 - 1.12)	0.75	(0.55 - 1.02)	0.58	(0.36 - 0.93)	0.02
Model 4	1.00	1.00	(0.78 - 1.29)	0.98	(0.72 - 1.35)	0.98	(0.62 - 1.55)	0.90
**δ-tocopherol**								
	Q1	Q2		Q3		Q4		Trend P
Person-years	14,109	14,250		14,832		14,726		
Number of cases	149	133		151		142		
Model 1	1.00	0.90	(0.71 - 1.14)	0.94	(0.75 - 1.18)	0.86	(0.68 - 1.09)	0.30
Model 2	1.00	0.89	(0.71 - 1.13)	0.94	(0.75 - 1.19)	0.84	(0.66 - 1.07)	0.23
Model 3	1.00	0.84	(0.66 - 1.08)	0.86	(0.67 - 1.12)	0.72	(0.52 - 0.99)	0.07
Model 4	1.00	0.91	(0.71 - 1.16)	0.97	(0.75 - 1.25)	0.88	(0.64 - 1.20)	0.52

Model 1: Adjusted for age and sex, and stratified by area.

Model 2: Multivariable model adjusted further for energy, smoking status, and DHA+EPA.

Model 3: Multivariable model adjusted further for variables in model 2 and α-linolenic acid.

Model 4: Multivariable model adjusted further for variables in model 2 and linoleic acid.
